# Functional analysis of *AIP* variants in a cohort of neuroendocrine neoplasms

**DOI:** 10.1530/ERC-25-0095

**Published:** 2026-01-07

**Authors:** Blanca R Carranza-Zavala, Julia M Zuarth-Vázquez, Susana M López-Zelocualtecatl, Claudia Ramírez-Rentería, Maribel Rodríguez-Torres, Ernesto Sosa-Eroza, Baldomero González-Virla, Armando Gamboa-Domínguez, Alfredo A Reza-Albarrán, Laura C Hernández-Ramírez

**Affiliations:** ^1^ Departamento de Endocrinología, Instituto Nacional de Ciencias Médicas y Nutrición Salvador Zubirán, Mexico City, Mexico; ^2^ Red de Apoyo a la Investigación, Coordinación de la Investigación Científica, Universidad Nacional Autónoma de México e Instituto Nacional de Ciencias Médicas y Nutrición Salvador Zubirán, Mexico City, Mexico; ^3^ Unidad de Investigación Médica en Enfermedades Endocrinas, Hospital de Especialidades, Centro Médico Nacional Siglo XXI, Instituto Mexicano del Seguro Social, Mexico City, Mexico; ^4^ Departamento de Endocrinología, Hospital de Especialidades, Centro Médico Nacional Siglo XXI, Instituto Mexicano del Seguro Social, Mexico City, Mexico; ^5^ Departamento de Patología, Instituto Nacional de Ciencias Médicas y Nutrición Salvador Zubirán, Mexico City, Mexico

**Keywords:** acromegaly, AIP, familial isolated pituitary adenoma, gigantism, neuroendocrine tumors

## Abstract

Loss-of-function (LOF) germline *AIP* variants are the main genetic cause of familial isolated pituitary adenoma and gigantism. A role for this defect in other neoplasms has been suggested, but remains unclear. We investigated the frequency, associated phenotypes, and *in vitro* functional effects of germline *AIP* variants in a cohort of Mexican patients with neuroendocrine neoplasms (NENs). Blood DNA samples from 101 adults (70.3% females) with isolated or syndromic NENs (50 with pituitary neuroendocrine tumors, PitNETs) were analyzed using a next generation sequencing panel. Targeted Sanger screening was carried out in additional family members and tumor samples. Missense and intronic variants were functionally assessed via cycloheximide chase assays or quantitative polymerase chain reaction plus sequence analysis of blood cDNA, as appropriate. Two rare likely benign defects (c.787 + 9C>T and p.T231M), two variants of uncertain significance (p.R106C and p.V291_L292del), one likely pathogenic (LP, p.C238Y), and one pathogenic (p.R304*) variant were found in six cases (5.9%). One individual was diagnosed with multiple gastric NENs and five carried PitNETs. Variant p.V291_L292del produced an unstable protein (*P* < 0.0001 for half-life curve, compared with wild type) and was reclassified to LP. Loss of heterozygosity in a gastric neuroendocrine tumor and nonsignificantly increased protein stability were observed for p.R106C. No deleterious effects were documented for c.787 + 9C>T. In conclusion, we determined the prevalence of *AIP* variants in a cohort of NENs and reclassified one VUS to LP. Our findings support the causal association of *AIP* LOF with PitNETs, but cannot rule out a role for *AIP* in other NENs.

## Introduction

Heterozygous inactivating germline *AIP* variants predispose individuals to the development of pituitary neuroendocrine tumors (PitNETs) within two different settings: familial isolated pituitary adenoma (FIPA) and early-onset PitNETs (1, 2). *AIP* variants cause 10–15% of cases of FIPA, an autosomal dominant disease with incomplete penetrance, in which two or more members of the same family develop PitNETs in the absence of other syndromic features (3, 4). FIPA-associated tumors are classically GH or GH/prolactin-secreting, large, invasive, with onset at an early age, male predominance, higher risk of pituitary apoplexy, and poor response to medical treatment (5, 6). Nevertheless, the clinical picture in individuals diagnosed due to targeted genetic screening might be indistinguishable from that of sporadic cases (4). Sporadic macroadenomas in patients under 18 years of age are due to germline *AIP* variants in up to 20% of cases, rising to 29–33% for pituitary gigantism (4, 6, 7, 8, 9, 10). Germline *AIP* variants are less common in adults with sporadic somatotropinomas (3–4%), while somatic *AIP* defects are extremely rare in such tumors (11, 12).

AIP is a co-chaperone protein with a vast list of interacting partners that can behave as a tumor suppressor or as an oncogene, depending on the cellular context (13). In the pituitary gland, AIP acts as a tumor suppressor, a function that is disrupted by PitNET-associated variants (5). Approximately 75% of pathogenic *AIP* variants result in a truncated and unstable protein with loss of important protein domains or in an absent protein due to nonsense-mediated RNA decay (5, 6, 14, 15). Multiple molecular consequences of AIP loss-of-function (LOF) have been described, including reduced Gi signaling and disruption of the interaction between AIP and phosphodiesterase-4A5 (PDE4A4) that lead to increased cyclic adenosine monophosphate (cAMP) signaling and downregulation of *ZAC1* expression and impairment of RET-induced apoptosis (16, 17, 18).

*AIP* overexpression has a pro-tumorigenic effect in extra-endocrine tumors, such as diffuse large B-cell lymphoma and colorectal cancer (13). Although early studies did not support a role for *AIP* LOF variants in the development of neoplasms other than PitNETs, various extra-pituitary tumors have been reported in *AIP* variant carriers, which could be coincidental or not (6, 19, 20). Interestingly, loss of heterozygosity (LOH) at pathogenic *AIP* variant loci in follicular thyroid carcinoma (FTC) and adrenocortical cancer samples from two individuals with concurrent diagnosis of FIPA have been documented (21, 22). Another instance of FTC with somatic LOH in the locus of a germline *AIP* variant within a FIPA kindred has been reported (23). Remarkably, this individual was not concurrently affected with a PitNET. Finally, an *AIP* variant of uncertain significance (VUS) was found in a patient with a prolactinoma and family history of primary hyperparathyroidism (PHPT), coexisting with a *MEN1* benign variant (24). Therefore, it is unclear whether *AIP* LOF could drive extra-pituitary neoplasia. In this study, we sought to characterize the clinical presentations associated with *AIP* variants in a cohort of Mexican patients with neuroendocrine neoplasms (NENs) and to verify their functional effects using *in vitro* assays.

## Materials and methods

### Patients and samples

The cases described here are part of a cohort of adult individuals with multiple types of NENs, including PitNETs. Participants were recruited at diagnosis or follow-up at either of two reference hospitals in Mexico City: Hospital de Especialidades de Centro Médico Nacional Siglo XXI, Instituto Mexicano del Seguro Social, and Instituto Nacional de Ciencias Médicas y Nutrición Salvador Zubirán. In selected cases, additional family members were also invited to participate. The protocol was approved by the Internal Review Boards of both institutions (protocol IDs R-2022-785-011 and 4090, respectively; ClinicalTrials.gov: NCT06523582), and all participants provided written informed consent. Peripheral blood DNA from 90 participants was analyzed by means of a custom 53-gene next generation sequencing panel (pNGS); these results have been published elsewhere (25). Eleven additional individuals were tested using an updated version of the pNGS with the addition of the *PAM* gene.

Single nucleotide variants (SNVs), indels, and structural *AIP* variants (NM_003977.4) with frequency <0.1% in the genome aggregation database v.4.0, the indigenous Mexican subset of samples from the Mexico City Prospective Study, and the All of Us databases were selected (26, 27, 28). When available, variant classification categories (according to the criteria of the American College of Medical Genetics and Genomics and Association for Molecular Pathology, ACMG/AMP) reported in ClinVar were noted (29). Frequency data, multiple *in silico* tools linked to the Varsome and Franklin online platforms, and previously published clinical and experimental data were used to classify variants with conflicting classification or not listed in ClinVar (30, 31).

Targeted Sanger sequencing was used to confirm pNGS results and to investigate *AIP* variants of interest (VOIs) in blood samples from additional family members and in formalin-fixed and paraffin-embedded (FFPE) tumor samples. Endpoint polymerase chain reaction (PCR) was carried out with GoTaq master mix (Promega, USA, M7122) and the following primers: 5′-CAG​CCC​ACG​GTG​ACA​GAG-3′ and 5′-GTT​CAC​GCA​TCT​GTG​CAA​CAC-3′ for a region of interest in exon 3, 5′-GGC​CCA​GGT​CTA​CAG​CTT​CTC-3′ and 5′-GGA​GAA​AGG​CCA​CTC​TCT​GAC-3′ for exon 5 and start of intron 5, and 5′-ATG​GTG​CCA​GGA​GAC​ATG​AG-3′ and 5′-CAG​AAG​CAT​GAC​GCA​GCA​C-3′ for exon 6. Amplicon sequencing was carried out at the Red de Apoyo a la Investigación molecular biology unit, using the forward primers and the BigDye Terminator 3.1 Cycle Sequencing Kit (Applied Biosystems 4337456) in a 3500xL Genetic Analyzer (Applied Biosystems, USA). Sequences were analyzed with the Geneious Prime v.2024.0.5 (Biomatters, Ltd, New Zealand) software.

### Expression plasmids

The pcDNA3.0-Myc-AIP plasmid (kindly donated by Prof. Márta Korbonits) was used as a template for site-directed mutagenesis to generate four variants, using either the Q5 Site-Directed Mutagenesis Kit (New England Biolabs, USA, E0552S) or the QuikChange II XL Kit (Agilent, USA, 200521) with the following primers: 5′-CAC​CGC​GAT​GTT​GCA​GAG​ACT​CTT​GGC​CA-3′ and 5′-TGG​CCA​AGA​GTC​TCT​GCA​ACA​TCG​CGG​TG-3′ for p.R106C, 5′-AGC​AGC​GGC​ATG​ATC​TGC​TGG​TCC​AGC-3′ and 5′-GCT​GGA​CCA​GCA​GAT​CAT​GCC​GCT​GCT-3′ for p.T231M, 5′-AGC​TGG​ACC​CAG​CCC​TGG-3′ and 5′CTT​TGG​CAA​AGT​CAG​CCT​GGG-3′ for p.V291_L292del, and 5′-CCC​GCA​GCT​CTC​AGC​TCA​CCA​CAG​G-3′ and 5′-CCT​GTG​GTG​AGC​TGA​GAG​CTG​CGG​G-3′ for p.R304*. The original plasmid, containing the full wild type human *AIP* coding DNA sequence (CDS), was used as a control. After sequence confirmation, purified plasmids were obtained using the GenElute HP Plasmid Maxiprep (Sigma-Aldrich, USA, NA0310-1KT).

### Experiments in cell lines

For cycloheximide (CHX) chase experiments, 2.5 × 10^5^ HEK293 cells (ATCC CRL-1573) were grown for 24 h in 12-well plates with minimum essential medium (Gibco, USA, 11095080) and 10% fetal bovine serum, at 37°C with 5% CO_2_. Cells were then transfected with 1 μg plasmid and 2 μL per well TurboFect (Thermo Scientific, USA, R0531) and treated with 25 μg/mL CHX (Sigma-Aldrich C4859-1ML) 24 h after transfection. At 0, 6, 12, and 24 h after treatment, cells were washed with 1× phosphate-buffered saline and lysed in 50 μL per well NP40 lysis buffer (Invitrogen, USA, FNN0021), supplemented with complete Protease Inhibitor Cocktail (Roche, Switzerland, 11697498001). Proteins were extracted by centrifugation and quantified with the Bradford method. All CHX chase experiments were done in triplicates and repeated at least three times for each condition.

### Western blot

For Western blot, 20 μg (CHX experiments) or 40 μg (comparative basal protein quantification) total proteins from each well were loaded into 15-well NuPAGE Bis–Tris Mini Protein Gels, 4–12% (Invitrogen NP0323BOX). After electrophoresis in 1× NuPAGE MES SDS Running Buffer (Invitrogen NP0002), proteins were transferred to nitrocellulose membranes and stained with Pierce Reversible Protein Stain Kit for Nitrocellulose Membranes (Thermo Scientific 24580), according to the manufacturer’s instructions. Membranes were blocked for 1 h with 0.5× Intercept (TBS) Blocking Buffer (LI-COR L03-92760003) and incubated overnight with 1:3,000 mouse anti c-Myc antibody (Sigma-Aldrich H3663-100UL). Goat anti-mouse IgG (Invitrogen A28177) at 1:5,000 was used as secondary antibody and Clarity Western ECL Substrate (Bio-Rad, USA, 1705060) was used for detection. Images were obtained after total protein staining (colorimetric protocol) and after chemiluminescent detection (chemi hi-resolution protocol) with the Image Lab 6.1.0 (Bio-Rad) software. Quantification was done by means of band densitometry; total proteins on each lane were used for normalization.

### Intronic variant assessment

Peripheral blood samples were obtained from one patient carrying an intronic *AIP* variant (Case 4) and four apparently healthy adult controls (two men and two women) on the same day, within 1 hour. RNA was extracted from all samples in parallel, using red cell lysis buffer (0.1 mM EDTA, 10 mM potassium bicarbonate, and 168 mM ammonium chloride) and the RNeasy Plus Mini Kit (QIAGEN, Germany 74134) plus DNase (QIAGEN 79254) treatment. The same kit was used to extract RNA from the HEK293 cell line. Reverse transcription of 500 ng total RNA from each sample was carried out using the SuperScript III First-Strand Synthesis SuperMix for qRT-PCR kit (Invitrogen 11752050). For quantitative PCR (qPCR), 10 ng coding DNA (cDNA) from blood RNA samples, no RNA and no reverse transcriptase controls, 1× TaqMan Fast Advanced Master Mix (Applied Biosystems 4444557), and 1× FAM-MGB-labeled *AIP* (Applied Biosystems Hs066610222_m1) or VIC-MGB-labeled *ACTB* control mix (Applied Biosystems 4325788) TaqMan assays were combined in a final volume of 10 μL. Three technical replicates per experimental condition were run in a StepOne (Applied Biosystems) instrument, and results were analyzed using the 2^−ΔΔCt ^method (32).

### Cloning

An 1,157 bp region containing the full-length *AIP* coding DNA sequence (NM_003977) was amplified using cDNA from HEK293 and peripheral blood cDNA from Case 4 with Q5 high-fidelity DNA Polymerase (New England Biolabs M0491S) and the primers 5′-GCT​TCT​GCC​CTC​AAC​CAA​AA-3′ and 5′-CAG​TGG​GCT​TGG​CAG​GTA​AG-3′. An 807 bp region was amplified likewise from peripheral blood DNA from Case 2, using the forward primer for *AIP* exon 5 and the reverse primer for exon 6. The amplicons were column-purified and cloned into pcDNA3.1/NT-GFP-TOPO, using the NT-GFP Fusion TOPO Expression Kit (Invitrogen K481001). Colony PCR was carried out with the primers included in the kit, and the amplicons were analyzed by agarose gel electrophoresis. Minipreps from multiple clones were produced (QIAprep Spin Miniprep Kit, QIAGEN 27104) and subjected to unidirectional forward sequencing.

### Statistical analyses

Analyses were carried out using Prism 10.4.1 (GraphPad Software, USA). Data distribution was analyzed with the Shapiro–Wilk test. Parametric data are presented as the mean ± standard deviation and non-parametric data are presented as the median and interquartile range (IQR). One-way ANOVA was used to compare basal protein expression for each variant against wild-type AIP. For CHX chase experiments, results were converted to percentages of the initial protein amount and half-life was calculated using a 1-phase decay equation for the summary of all experiments for wild-type *AIP* and each variant studied. Half-life curves were compared among the experimental conditions using two-way ANOVA; correction for multiple comparisons was done with the Dunnett test. A Kruskal–Wallis test with Benjamini, Krieger, and Yekuteli *post-hoc* correction for multiple comparisons was used to compare the qPCR results among samples. *P* < 0.05 was considered statistically significant.

## Results

### *AIP* variants in the context of the study cohort

We screened 101 individuals (70.3% females, *n* = 71) with age at disease onset of 37.1 (20.5–49.8) years and age at diagnosis of 38.8 (23–51) years. Clinical diagnoses encompassed 17 categories: ectopic Cushing’s syndrome (*n* = 2), early-onset (≤30 years) GH excess (gigantism or acromegaly, *n* = 10) or other (non-GH-secreting) PitNETs (*n* = 19), multiple endocrine neoplasia (MEN)-like presentations (*n* = 13), MEN1 (*n* = 15), MEN2A (*n* = 3), MEN2B (*n* = 1), medullary thyroid carcinoma (*n* = 8), multiple gastric NENs (*n* = 2), neurofibromatosis type 1 (NF1, *n* = 1), PHPT (*n* = 1), PitNET with onset >30 years (*n* = 5), single (*n* = 5) or multiple pancreatic NENs (*n* = 1), single (*n* = 2) or multiple paragangliomas or pheochromocytomas (*n* = 10), and von Hippel–Lindau syndrome (*n* = 3).

Fifty probands (49.5%) carried PitNETs, out of which 32% (*n* = 16) were GH-producing tumors, 28% (*n* = 14) were corticotropinomas, 22% (*n* = 11) were NF-PitNETs, and 18% (*n* = 9) were prolactinomas. Presentation was syndromic in one-third (*n* = 16) of participants, including cases of MEN1 (*n* = 12), MEN-like (*n* = 3), and NF1 (*n* = 1). There was a relevant family history in 46% (*n* = 23) of probands with PitNETs, including kindreds with cancer predisposition (*n* = 10), FIPA (*n* = 9), MEN-like (*n* = 2), MEN1 (*n* = 1), and NF1 (*n* = 1).

Six *AIP* VOIs, all of them SNVs or indels, were identified in the cohort; no structural variants were found. Two VOIs were detected in the same individual, one variant was found in two different cases, and three other participants carried a different variant each, accounting for six different individuals (5.9% of those screened); these cases are detailed below.

### Case 1

This lady with past history of bilateral breast fibroadenomas presented at age 53 years with dysphagia due to esophageal stenosis, requiring repeated dilatations. Within 2 years from her initial presentation, two gastric adenomatous polyps were endoscopically resected. Between ages 56 and 70 years, a total of five more gastric polyps (2–4 mm in their maximum diameter) were removed. In contrast with the initial lesions, three tumors were diagnosed as neuroendocrine neoplasms, of which two were categorized as well-differentiated neuroendocrine tumors (NETs). No metastases were documented, but due to disease recurrence, she was started on lanreotide (90 mg every 6 weeks) at age 71 years. During her clinical evolution, she developed type 2 diabetes mellitus, multiple additional gastric adenomatous polyps, *H. pylori*-associated intestinal metaplasia, and primary hypothyroidism with positive antibodies, atrophic gastritis with hypergastrinemia and pernicious anemia. Based on these data, a diagnosis of type 1 gastric NENs was established. None of her relatives had a relevant medical history.

### Case 2

At age 14 years, this lady developed headaches, secondary amenorrhea, prognathism and acral growth, which led to a diagnosis of acromegaly due to a pituitary macroadenoma 1 year later. She was treated with bromocriptine for 5 years and tamoxifen for 3 years, with partial improvement. Her symptoms eventually worsened, and at age 30 years, a transsphenoidal surgery was carried out, resecting a somatotropinoma (GH+, ACTH−, and prolactin−). Because of a residual lesion (4.7 × 3.4 mm), her disease remained active and required additional treatment with fractioned radiotherapy (54 Gy). At age 51 years, she remains in remission and receives treatment for secondary hypopituitarism, secondary diabetes mellitus, trigeminal neuralgia, and narrow cervical, thoracic, and lumbar canal. One of her maternal first cousins has a history of microprolactinoma.

### Case 3

**Figure 1:**
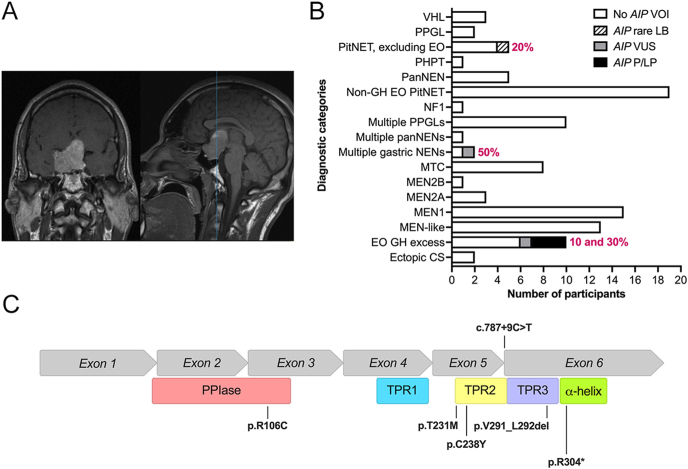
Initial assessment of *AIP* variants in the study cohort. (A) Coronal (left) and sagittal (right) representative gadolinium-enhanced T1-weighted preoperatory magnetic resonance images of Case 3, showing a pituitary macroadenoma. (B) Frequency of *AIP* variants within each clinical category included in the cohort of adult patients with NENs. (C) Schematic representation of *AIP* exons (top) and corresponding protein domains (bottom) displaying the VOI identified in this study. α-helix, carboxy-terminal alpha-helix; CS, Cushing’s syndrome; EO, early onset; GH, growth hormone; LB, likely benign; MEN, multiple endocrine neoplasia; MEN1, MEN type 1; MEN2A and MEN2B, MEN types 2A and 2B; MTC, medullary thyroid carcinoma; NENs, neuroendocrine neoplasms; NF1, neurofibromatosis type 1; P/LP, pathogenic and likely pathogenic; PanNEN, pancreatic neuroendocrine tumor; PHPT, primary hyperparathyroidism; PitNET, pituitary neuroendocrine tumor; PPGL, pheochromocytoma or paraganglioma; PPIase, peptidyl-prolyl cis–trans isomerase domain; TPR1, TPR2, and TPR3, tetratricopeptide repeats 1, 2, and 3; VHL, von Hippel–Lindau syndrome; VOI, variants of interest; VUS, variant of uncertain significance.

At age 14 years, this young man developed accelerated growth, coarse facial features, prognathism, acral growth, and headaches. Four years later, he was diagnosed with gigantism (height 3.4 SD over midparental height) due to a pituitary macroadenoma (44 × 51 × 48 mm, Knosp 4, Fig. 1A). By then, he had developed bitemporal hemianopsia, diabetes mellitus, dyslipidemia, high blood pressure, colon polyps and hypopituitarism (central hypocortisolism, hypogonadotropism, and hypothyroidism). He received octreotide LAR 20 mg monthly for 4 months and underwent transsphenoidal surgery at age 22 years. Unfortunately, the tumor resection was incomplete due to profuse bleeding and involvement of the right cavernous sinus. Since disease activity persisted postoperatively, he was started on lanreotide 120 mg monthly and cabergoline 2 mg/week. Total thyroidectomy was carried out 1 year later due to a 17 mm thyroid imaging reporting and data system (TI-RADS) 5 and Bethesda V nodule. This lesion was ultimately diagnosed as thyroid follicular nodular disease with a 2 cm adenomatous nodule on the right lobe. Radiotherapy to the sellar region was planned, but by age 24 years, the patient had been lost from follow-up. His family history was unremarkable.

### Case 4

This woman was diagnosed with acromegaly due to a pituitary microadenoma at age 38 years. She referred a 3-year history of acral growth, arthralgias and paresthesias in both hands, coarse facial features and oligomenorrhea. The patient underwent transsphenoidal surgery at age 43 years without achieving remission. Postoperatively, she was treated with octreotide LAR, eventually developing tumor recurrence. A transsphenoidal reintervention was aborted before tumor resection due to intraoperative bleeding. Therefore, fractioned radiotherapy to the sellar region (54 Gy) was subsequently carried out. The patient persisted with active disease, and a tumor remnant in the sphenoid sinus was identified by magnetic resonance imaging at age 48 years. Biochemical control was ultimately achieved by treatment with lanreotide 90 mg monthly and cabergoline 1 mg weekly. At age 50 years, she requires hormone replacement for her central hypogonadism, hypothyroidism, and hypocortisolism, and treatment for mixed dyslipidemia and osteoporosis with idiopathic hypercalciuria. The patient noticed acromegaloid features in her father, but he was not clinically evaluated and has since passed away.

### Case 5

This 52-year-old gentleman first presented around age 15 years with accelerated growth, headaches, photophobia, acral growth, and decreased libido. He was diagnosed with gigantism due to a pituitary macroadenoma (10 × 10 mm) at age 18 years and underwent transsphenoidal surgery soon after. Since tumor resection was incomplete, his hyposomatotropinemia persisted postoperatively, requiring fractioned radiotherapy. He eventually achieved remission, although at the expense of hypopituitarism. Over the course of his disease, the patient also developed secondary diabetes mellitus, mixed dyslipidemia, non-alcoholic fatty liver disease, cardiac sinus dysfunction requiring a pacemaker, and colon polyps. His paternal family history included one first cousin with confirmed acromegaly and a history of craniotomy for an indication unknown to the patient in the son of a different first cousin, who passed away after the surgery.

### Case 6

This 29-year-old young man first noticed headaches and acral growth around age 18 years. Four years later, he was diagnosed with acromegaly due to a pituitary macroadenoma (19 × 21 × 21 mm) and underwent transsphenoidal surgery, without achieving remission. Treatment with lanreotide 120 mg every 6 weeks and cabergoline 1.5 mg weekly was then started and radiosurgery (20.6 Gy) was performed at age 23 years. The patient developed central hypogonadism, and during the course of his disease, a TI-RADS 2 thyroid nodule, and mild tricuspid insufficiency were also detected. Over the past 6 months, the doses of lanreotide and cabergoline were progressively reduced, and at age 29 years, he remains with normal IGF-1 under treatment with only lanreotide 120 mg every 2 months. His family history is remarkable for the diagnosis of acromegaly in two distant cousins on the maternal side.

### Assessment of variant pathogenicity

The following germline VOIs were identified in our cohort: p.R106C in Case 1, p.T231M and p.V291_L292del in Case 2, p.C238Y in Case 3, c.787 + 9C>T in Case 4, and p.R304* in Cases 5 and 6 (Fig. 1B and Table 1). Out of these variants, c.787 + 9C>T and p.T231M were classified as LB, p.R106C and p.V291_L292del as VUS, p.C238Y as likely pathogenic, and p.R304* as pathogenic. The VUS p.R106C, in exon 3, changes a residue in the peptidyl-prolyl cis–trans isomerase domain of AIP, while the rest of variants, located within exon 5, intron 5, and exon 6, affect the C-terminal region (Fig. 1C).

**Table 1:** Clinical associations, functional analyses and classification of *AIP* variants identified in the study.

	Effect and location in gene	Allele frequency in population databases (%)	Reported clinical and experimental data or *in silico* analyses	Clinical associations in this study	ACMG/AMP classification
Variant* (dbSNP ID)					
c.316C>T, p.R106C (rs369414668)	Missense, exon 3	All of us: 0.0039, gnomAD: 0.0075, MCPS IMX: 0.0027	Germline defect in one case of prolactinoma (male, diagnosis age 31 y), coexisting with *MEN* (NM_130799.3) VUS p.R171P (24). Three reports in ClinVar (undisclosed diagnosis, somatotropinoma, and hereditary cancer-predisposing syndrome). Maps to an HSP90 binding site; deleterious effect predicted by multiple *in silico* tools. Individual tools: AlphaMissense: likely pathogenic, 0.724; DANN: deleterious, 1; polyphen2: probably damaging, 1; FATHMM: damaging, −4.73; MutationTaster: disease causing, 1; PROVEAN: damaging, −7.34. Meta scores: CADD: score = 34; BayesDel: deleterious moderate, 0.31; MetaLR: deleterious, 0.96; REVEL: deleterious moderate, 0.895	Germline variant in one patient with multiple gastric NENs (Case 1)	VUS (VCV000485059.10)
					Our study: VUS
c.692C>T, p.T231M (rs532170807)	Missense, exon 5	All of us: 0.0061, gnomAD: 0.0039, MCPS IMX: 0.1505	Found at the germline level in four cases reported in ClinVar (undisclosed diagnosis in two cases, somatotropinoma, and hereditary cancer-predisposing syndrome)	Germline variant in a patient with early-onset acromegaly from a FIPA kindred, coexisting with germline *AIP* VUS and somatic LB variant (Case 2)	Conflicting interpretations of pathogenicity: VUS, benign, LB (VCV000759986.16). Our study: LB
c.713G>A, p.C238Y (rs267606569)	Missense, exon 5	gnomAD: 0.0001, all of us and MCPS IMX: n/a	Cosegregation with PitNETs in one FIPA kindred (2). Two reports in ClinVar (undisclosed diagnosis and hereditary cancer-predisposing syndrome). Unstable protein, loss of multiple molecular interactions (2, 14, 18, 33, 41)	Germline variant in one patient with apparently sporadic gigantism (Case 3)	ClinVar: LP (VCV000041197.9)
					Our study: LP
c.787 + 9C>T, p.? (rs749392143)	Intronic, intron 5	All of us: 0.0082, gnomAD: 0.0045, MCPS IMX: n/a	Reported as a germline variant in one case of sporadic early-onset somatotropinoma (6). Two additional reports in ClinVar (undisclosed diagnoses). *In silico* analyses with multiple tools predict no deleterious effect	Germline variant in one patient with acromegaly and suspected FIPA, also carrying the *FH* (NM_000143.4) p.K477dup VUS (Case 4). Somatic variant in one case of early-onset acromegaly from a FIPA kindred, coexisting with one germline *AIP* LB variant and one germline VUS (Case 2)	LB (VCV001107627.8)
					Our study: LB
c.872_877del, p.V291_L292del	In-frame deletion, exon 6	All of us, gnomAD, and MCPS IMX: n/a	Germline variant in one Mexican individual with apparently sporadic gigantism. Predicted to disrupt the packaging of the C-terminal α-helix (35)	Germline variant in one case of early-onset acromegaly from a FIPA kindred, coexisting with germline and somatic *AIP* LB variants (Case 2). One apparently unaffected variant carrier identified	ClinVar: n/a
					Our study: VUS (changed to LP after functional assessment)
c.910C>T, p.R304* (rs104894195)	Nonsense, exon 6	All of us: 0.0007, gnomAD: 0.0014, MCPS IMX: n/a	Most frequent germline defect causing FIPA (4). Leads to a truncated protein with loss of multiple molecular interactions and shortened half-life (33, 41)	Germline variant in two FIPA families with GH-producing PitNETs only (Cases 5 and 6). Five apparently unaffected variant carriers (four tested and one obligate carrier) in one family (Case 5) and three (all tested) in the other family (Case 6)	Pathogenic (VCV000004888.33)
					Our study: pathogenic

* Variants are presented in Human Genome Variation Society nomenclature, according to the reference sequence NM_003977.4.

FIPA, familial isolated pituitary adenoma; gnomAD, gnomAD v.4.1.0 exomes + genomes; LB, likely benign; LP, likely pathogenic; MCPS IMX, Mexico City Prospective Study, indigenous Mexican; n/a, not available; NENs, neuroendocrine neoplasms; PitNETs, pituitary neuroendocrine tumors; VUS, variant of uncertain significance; y, years.

Cases 3, 5, and 6, carrying pathogenic or likely pathogenic (P/LP) variants, accounted for 30% of the cases of early onset GH excess in the cohort (ten in total), while Case 2, carrying two germline VUS and one somatic LB variant (detailed below), made up for a further 10% of the patients within the same diagnostic category. Case 4, carrying a LB variant, was one of five participants with isolated PitNETs with onset at age >30 years. This patient also carried the germline *FH* (NM_000143.4) VUS p.K477dup; no other clinically relevant variants were found in the rest of individuals carrying *AIP* VOIs. Case 1, carrying a VUS, was one out of two participants in the cohort with diagnosis of multiple gastric NENs and the only carrier of an *AIP* VOI not diagnosed with a PitNET. A targeted clinical evaluation revealed no signs or symptoms indicative of such diagnosis, and her basal cortisol, FSH, GH, IGF-1, LH prolactin, and TSH were within the normal ranges for her age and sex.

### Genetic screening in additional family members and tumor samples

**Figure 2:**
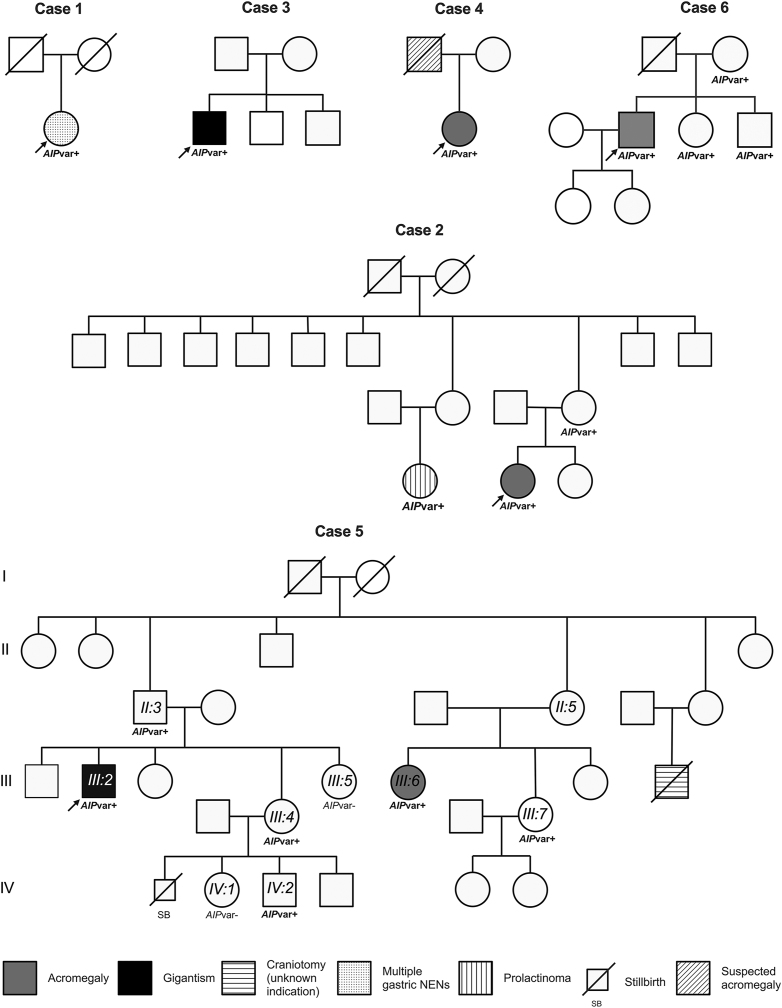
Pedigrees of families with *AIP* VOI. *AIP*var+, *AIP* variant carrier; *AIP*var−, confirmed not carrier of *AIP* variant.

Pedigrees for the six cases of interest are presented in Fig. 2. Aside from the probands, targeted genetic screening was carried out in two relatives of Case 2, seven relatives of Case 5, and three relatives of Case 6. The apparently healthy mother (age 78 years) and the affected first cousin of Case 2 are both variant carriers. In the family of Case 5, two out of seven variant carriers (28.6%, including one obligate carrier) are known to be affected. Individual II:3 (the proband’s father) is a variant carrier and remains apparently unaffected at age 76 years; one of his sisters (II:5) is an obligate carrier. Two other variant carriers, III:4 and IV:2, are currently 47 and 19-years-old and declined imaging and biochemical screening for PitNETs. Individual IV:2 referred frequent headaches starting at age 15 years, but no other relevant symptoms. Remarkably, individual III:7 is 12 cm taller than the average height of her two sisters. At age 38 years, she refers no symptoms of concern and has not yet undergone clinical screening. Likewise, the mother and two siblings of Case 6 are variant carriers, but have not been diagnosed with a PitNET. The patient’s mother (age 50 years) is asymptomatic, his 27-year-old sister refers menstrual disturbances, but has not yet been formally evaluated, and his 20-year-old brother is slightly below the midparental height and has no symptoms of concern. There were no individuals available for testing in the rest of families.

**Figure 3:**
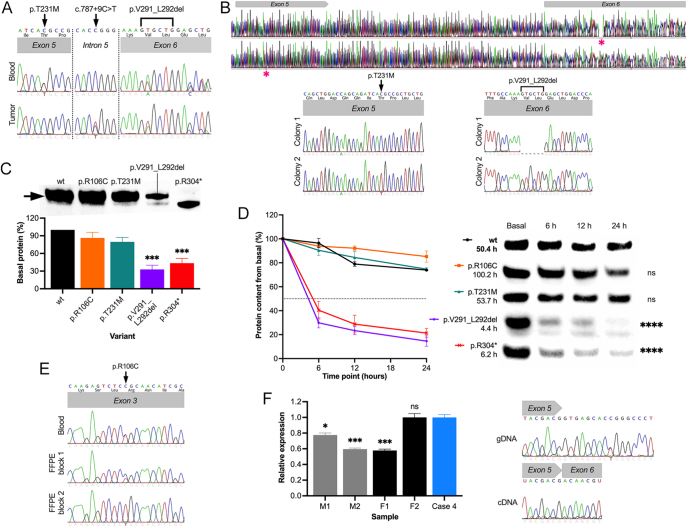
Functional assessment of *AIP* variants. (A) Sanger sequencing results of blood (top) and formalin-fixed and paraffin-embedded (bottom) samples from Case 2. Heterozygous variants p.T231M and p.V291_L929del were identified in the blood sample, while tumor sample showed loss of p.T231M, preserved heterozygosity of p.V291_L929del, and the heterozygous somatic intronic variant c.787 + 9C>T. (B) Phasing analysis of the two germline variants detected in Case 2. Via TOPO-TA cloning and Sanger sequencing, we found that variants p.T231M and p.V291_L929del are in *trans* in peripheral blood DNA. Results for two representative colonies are shown. (C) Wild-type AIP and VOI, overexpressed in HEK293 cells and detected by Western blot (top). Bands for all variants, except p.R304*, were observed at 37 kDa (arrow). Quantification showed significantly reduced protein expression for p.V291_L292del and p.R304*, compared with wild-type AIP (bottom). (D) Half-life curve graph (left) and representative Western blot images (right) of the cycloheximide chase experiments for wild-type AIP and the VOI. A significantly reduced half-life was observed for the VUS p.V291_L292, compared with the wild-type protein. Similar results were obtained for the pathogenic variant p.R304*, as previously reported. The variant p.R106C displayed longer half-life than the wild-type protein, although this difference did not reach statistical significance. (E) Sanger sequencing results of blood (top) and two blocks (bottom) of a formalin-fixed and paraffin embedded (FFPE) gastric NET from Case 1. LOH at the variant locus is observed in one of samples (FFPE block 2). (F) Evaluation of the intronic variant c.787 + 9C>T by quantitative PCR (left) and Sanger sequencing (right). *AIP* expression in peripheral blood cDNA was significantly lower in three out of four apparently healthy controls, compared with Case 4. Under our experimental conditions, this heterozygous intronic variant did not result in alternative transcripts. ***, *P* < 0.001; ****, *P* < 0.0001; cDNA, coding DNA; gDNA, genomic DNA; h, hours; M1, male control 1; M2, male control 2; F1, female control 1; F2, female control 2; ns, not significant; wt, wild type.

DNA from pituitary tissue was available for Case 2 only. This sample displayed absence of p.T231M and preserved heterozygosity for p.V291_L292del, but harbored the heterozygous variant c.787 + 9C>T, which was absent from the patient’s blood sample (Fig. 3A). In this patient, phasing analysis showed that variants p.T231M and p.V291_L292del are in *trans* at the germline level (Fig. 3B).

### Functional assays

We analyzed the effects of two AIP VUS p.R106C and p.V291_L292del on the AIP protein. Variant p.T231M (LB) and p.R304* (pathogenic) were also included for comparison. In basal conditions, overexpression of AIP p.V291_L292del and p.R304* resulted in a significantly reduced amount of detectable protein (32.9 ± 12.4% and 43.5 ± 14.1% of wild type, respectively, *P* = 0.0002 and 0.0009, Fig. 3C). In contrast, overexpression of variants p.R106C and T231M produced similar amounts of protein, compared with wild-type AIP (86.3 ± 16.7% and 79.7 ± 13.1%, respectively, *P* = 0.5147 and 0.2158).

Protein stability was assessed using a previously validated CHX chase assay (33). In concordance with basal protein expression, this assay demonstrated a significantly shortened half-life for p.V291_L292del (4.4 h) compared with the wild-type protein (50.4 h, *P* < 0.0001 for half-life curves, Fig. 3D). The half-life curve of this in-frame deletion was similar to that of the previously known unstable protein p.R304* (6 h in this study, *P* < 0.0001 compared with wild type and *P* = 0.5728 compared with p.V291_L292del). Conversely, the half-life curve of p.T231M showed a stable protein and was nearly identical to that of wild-type AIP (53.7 h, *P* > 0.9999).

Interestingly, p.R106C displayed the longest half-life of all analyzed variants (100.2 h), although the comparison with wild-type AIP did not reach statistical significance (*P* = 0.4268). We analyzed the status of the germline variant p.R106C in a gastric well-differentiated NET from Case 1 (Fig. 3E). Preserved heterozygosity with predominance of the reference allele was found in one block of tissue, while a second block displayed clear absence of the reference nucleotide.

Finally, the intronic variant c.787 + 9C>T, located out of canonical splicing sites, was detected in two different cases, either as a somatic (Case 2) or as a germline (Case 4) defect. This variant is quite infrequent in the general population and has been reported in association with early onset acromegaly in a single individual (6). We decided to evaluate this variant *in vitro* because the analysis of intronic changes out of canonical splice sites by *in silico* methods might not be ideal (34). Three out of four controls displayed reduced *AIP* RNA expression compared with Case 4 (relative expression 0.98 (0.89–1.1) for Case 4 and 0.75 (0.70–0.83), 0.60 (0.58–0.63), 0.59 (0.52–0.62), and 0.98 (0.89–1.2) for controls M1, M2, F1, and F2, respectively; *P* = 0.0334, <0.0001, <0.0001, and 0.9162). Colony PCR of plasmids containing the full *AIP* CDS from Case 4 showed no additional amplicons, compared with HEK293 controls. Concordantly, sequencing of the *AIP* CDS PCR product and five different clones of plasmids showed no differences in the exon 5–6 junction, compared with the reference. These data do not support a functional effect for the intronic variant found in our study.

## Discussion

Following the discovery of germline heterozygous *AIP* LOF variants as drivers of FIPA, a causative association with other neoplasms was sought, rendering negative results (19, 20). Later, other studies suggested a possible role for *AIP* LOF in PitNETs coexisting with other endocrine tumors in a single patient or family (21, 22, 24). These findings, along with the recently described protumorigenic effect of *AIP* in cancer, prompted us to reassess whether germline *AIP* variants could be involved in extra-pituitary NENs. In a previous study in the Mexican population, we reported *AIP* VOIs in 7% of cases of young-onset GH excess (35). We have now identified six *AIP* VOIs in an equal number of individuals from our heterogeneous cohort of individuals with NENs, accounting for 5.9% of the total participants. Aside from one individual with multiple gastric NENs, all variant carriers presented with isolated PitNETs and had no personal or family history indicative of syndromes of multiple endocrine neoplasia.

Upon detection, two variants (c.787 + 9C>T and p.T231M) were classified as LB and two (p.R106C and p.V291_L292) were assigned to the VUS category, while the remaining two were considered LP (p.C238Y) or pathogenic (p.R304*), according to the ACMG/AMP guidelines. VUSs are a frequent result of diagnostic genetic tests that may entangle decisions on disease management and genetic counseling, due to their inconclusive nature (36). Multiple recent studies have successfully reclassified VUS, but the growing use of clinical genetic testing has also increased the number of findings requiring further evaluation (37). Particularly for *AIP*, the incomplete penetrance of FIPA and the involvement of this gene in many different cellular processes make variant assessment a complex task (38). Nevertheless, reclassification of *AIP* variants has previously been achieved through a combination of detailed clinical information, cosegregation data, additional somatic and germline genetic analyses, *in silico* predictions, and *in vitro* functional assays (39).

The in-frame deletion p.V291_L292del is absent from the most recent versions of the gnomAD and all of us datasets. It is not reported within the indigenous Mexican population of MCPS either, although it has a frequency of 0.0071% in the European subgroup of the same database. One case of sporadic gigantism from our previous study in the Mexican patients with PitNETs accounts for the only clinical association reported for this variant (35). This individual is apparently unrelated to Case 2, but it was not possible to re-contact him for the present study. Cosegregation of the variant with a PitNET was demonstrated in the family of Case 2, although with the characteristic incomplete penetrance of *AIP*-associated FIPA. The probability of a phenocopy, however, cannot be fully ruled out, since microprolactinomas are common in the general population. Our experimental approaches allowed for the reclassification of this indel from VUS to LP (ACMG/AMP criteria met: PS3, PM2, PM4, and PP3). In contrast, the remaining variants evaluated remained within their original clinical categories.

Intriguingly, the VUS p.R106C displayed increased protein stability compared with wild-type AIP, replicating a degradation pattern previously observed for other missense variants (p.R16H, p.M170T, p.R304Q, and p.R325Q) (33). Nevertheless, in none of these cases was the difference with the wild-type protein statistically significant. Three of these variants (p.R16H, p.R304Q, and p.R325Q) are currently classified as benign or LB, while the remaining (p.M170T), reported in a single case, remains as a VUS (4, 8, 39). Therefore, the pathophysiological relevance of an increased *AIP* half-life, if any, is unclear. Besides, the patient carrying this variant was not diagnosed with a PitNET, in contrast with the rest of carriers of VOIs. Intriguingly, however, one gastric NET from this individual showed absence of the reference allele at the variant locus. Although not a universal finding, LOH is usually considered to support pathogenicity for variants in tumor suppressor genes. With the available data, we cannot support or rule out a role of *AIP* as a driver of gastric NENs, either due to increased protein stability or via LOF.

The limitations of our study include the low availability of tumor samples, the small number of individuals genetically screened within most kindreds, and the lack of data on the targeted clinical screening of variant carriers. The current expert recommendations support the biochemical and imaging screening of *AIP* P/LP variant carriers, since early diagnosis of PitNETs in this setting positively impacts the clinical outcomes (4, 15). Unfortunately, in our country, the low availability of genetic tests and the limited experience of most health professionals with the use of genetic data jeopardizes the establishment of strategies for the routine follow-up of variant carriers (40). Other barriers, such as the economic burden of screening tests, the anxiety associated with the variant carrier status, and the incomplete disease penetrance, might also play a role. Finally, our choice of functional assays was based on previous research. Unfortunately, these methods cannot accurately assess the effects on the multiple cellular processes in which AIP is involved.

In summary, our data support the established notion that germline *AIP* LOF variants are causally associated with PitNETs, while the role of *AIP* in other NENs is not yet well defined. Our experimental approaches demonstrated that p.V291_L292del results in an unstable AIP protein and p.R106C has unclear functional impact, while c.787 + 9C>T and p.R106C do not seem to entail deleterious effects. Based on our results, we reclassified p.V291_L292del from VUS to LP, which will hopefully improve the future clinical management and genetic counseling of individuals carrying this genetic defect.

## Declaration of interest

The authors declare that there is no conflict of interest that could be perceived as prejudicing the impartiality of the work reported.

## Funding

Our work was supported by departmental funding from the Coordination of Scientific Research from Universidad Nacional Autónoma de México (UNAM), research grants from UNAM’s Support Program for Research Projects and Technological Innovation (UNAM-PAPIIT, project TA200524) and Sociedad Mexicana de Nutrición y Endocrinología, and an equipment grant from the Society for Endocrinology.
